# Determining carnivore habitat use in a rubber/forest landscape in Brazil using multispecies occupancy models

**DOI:** 10.1371/journal.pone.0195311

**Published:** 2018-04-16

**Authors:** Andrea Dechner, Kevin M. Flesher, Catherine Lindell, Téo Vega de Oliveira, Brian A. Maurer

**Affiliations:** 1 Department of Fisheries and Wildlife, Michigan State University, East Lansing, Michigan, United States of America; 2 Program in Ecology, Evolutionary Biology and Behavior, Michigan State University, East Lansing, Michigan, United States of America; 3 Centro de Estudos da Biodiversidade, Reserva Ecológica Michelin, Igrapiúna, Bahia, Brasil; 4 Department of Integrative Biology, Michigan State University, East Lansing, Michigan, United States of America; 5 Center for Global Change and Earth Observations, Michigan State University, East Lansing, Michigan, United States of America; 6 Divisão de Mamíferos do Museu de Zoologia, Departamento de Ciências Biológicas, Universidade Estadual de Feira de Santana, Feira de Santana, Bahia, Brasil; 7 Center for Statistical Training and Consulting, Michigan State University, East Lansing, Michigan, United States of America; Liverpool John Moores University, UNITED KINGDOM

## Abstract

Understanding the factors that influence the presence and distribution of carnivores in human-dominated agricultural landscapes is one of the main challenges for biodiversity conservation, especially in landscapes where setting aside large protected areas is not feasible. Habitat use models of carnivore communities in rubber plantations are lacking despite the critical roles carnivores play in structuring ecosystems and the increasing expansion of rubber plantations. We investigated the habitat use of a mammalian carnivore community within a 4,200-ha rubber plantation/forest landscape in Bahia, Brazil. We placed two different brands of camera traps in a 90-site grid. We used a multispecies occupancy model to determine the probabilities of habitat use by each species and the effect of different brands of camera traps on their detection probabilities. Species showed significant differences in habitat use with domestic dogs (*Canis familiaris*) and crab-eating foxes (*Cerdocyon thous*) having higher probabilities of using rubber groves and coatis (*Nasua nasua*) having a higher probability of using forest. The moderate level of captures and low detection probabilities (≤ 0.1) of tayras (*Eira barbara*) and wildcats (*Leopardus* spp.) precluded a precise estimation of habitat use probabilities using the multispecies occupancy model. The different brands of camera traps had a significant effect on the detection probability of all species. Given that the carnivore community has persisted in this 70-year-old landscape, the results show the potential of rubber/forest landscapes to provide for the long-term conservation of carnivore communities in the Atlantic forest, especially in mosaics with 30–40% forest cover and guard patrolling systems. The results also provide insights for mitigating the impact of rubber production on biodiversity.

## Introduction

Determining how wildlife respond to landscape modifications is one of the principal goals of conservation ecology, particularly if we aim to devise effective land management strategies to ensure their long-term persistence in human-dominated landscapes. Responses of mammalian carnivores to agricultural lands vary, depending not only on the type of cultivar and the amount and configuration of native vegetation remaining in the matrix, but also on the ecology of each species [1–3]. Species responses to land conversion resulting in differential habitat use may modulate intra/interspecific interactions, and thus influence the effect of the species on other tropic levels [4–6]. Given that predators play critical roles in ecosystem structure and function [7], devising land management strategies for carnivore conservation in agricultural landscapes requires investigations of habitat use on each carnivore species in the community.

Tropical rainforests are among the most species-rich and threatened ecosystems on the planet, with vast areas being converted to agricultural lands each year [8]. The demand for natural rubber (*Hevea brasiliensis*) is one of the principal drivers of forest conversion in some of the most species-rich rainforests, with more than 2 million ha planted globally during the past decade [9]. Only in mainland southeast Asia, an additional 4.3–8.5 million ha are expected to be planted by 2024 [9]. Given this situation, it is essential to determine how carnivores use rubber plantation landscapes and whether land management strategies can be devised to accommodate these species. Although previous studies have documented carnivore habitat use within tropical agricultural landscapes, none has done so in a rubber/forest mosaic and within a multispecies occupancy modeling framework.

The majority of natural rubber is produced in Southeast Asia, but rubber is also grown in West Africa and throughout Central and South America. Although the rubber tree is native to Brazil, the prevalence of South American leaf blight caused by the endemic fungus (*Microcyclus ulei*), impedes this country from producing sufficient rubber to meet its needs. Nonetheless, the federal government has the goal of making the nation self-sufficient in natural rubber by increasing the area under cultivation [10]. The majority of the internal production of rubber is located in the Atlantic forest [10], a biodiversity hotspot of which, after 500 years of intensive human exploitation, less than 16% remains, mostly in fragments <50 ha [11]. Some of the first rubber plantations in the Atlantic forest were planted in the state of Bahia in the 1950s by converting extensive tracts of lowland rainforest into mosaics of forest and rubber groves. After seven decades, these plantations offer an opportunity to study the habitat use of carnivores in the resulting mosaic, and whether rubber plantations can be managed to allow for carnivore persistence while remaining economically viable.

The aim of this study was to determine how a carnivore community in one of the oldest rubber plantations in Bahia utilizes the resulting landscape mosaic, using occupancy models to test for differential habitat use by the species while accounting for their detection probability. In addition, given the potential influence of sampling tools on the probability of detecting species [12–14], we assessed the effect of different brands of camera traps on the detection probability of carnivore species.

To our knowledge this is the first study on the ecology of a neotropical carnivore community in a rubber/forest landscape. This type of work is essential as rubber is an agricultural commodity whose effects on biodiversity are poorly known in any of the tropical regions where it is grown [9, 15]. The results provide insights to help guide managers of old and new plantations throughout the tropics about how to accommodate wildlife and mitigate the impact of rubber on biodiversity.

## Methods

### Study site

**Fig 1:**
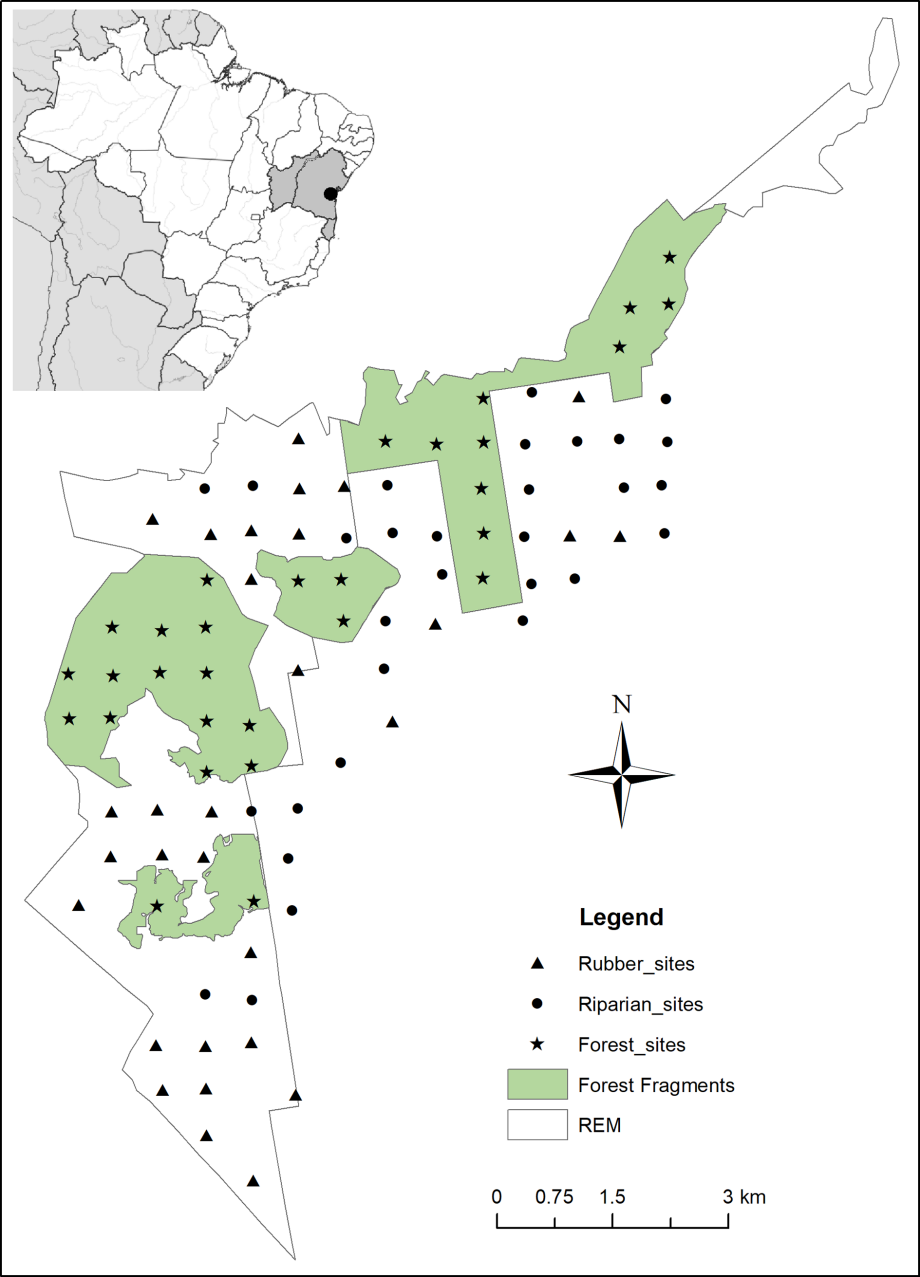
Study area and location of the sampling sites, Ituberá/Igrapiúna, Bahia, Brazil, 2013–2014.

The 4,200-ha study site included the 3,386 ha of the Reserva Ecológica Michelin (REM) and adjacent properties in the municipalities of Ituberá and Igrapiúna, Bahia, Brazil (39°10’W 13°47’S to 39°13’W 13°54’S) (Fig 1). The study area is embedded in a regional landscape that retains approximately 40% forest cover and is part of a 9,000-ha rubber plantation established by Firestone in 1950s. The natural vegetation is lowland evergreen broadleaf moist forest. Average annual rainfall is about 2,000 mm with no distinct dry season [16]. Monthly rainfall ranges between 118 mm and 208 mm. Minimum average monthly temperatures range between 18.6 C and 21.9 C, while the maximum average monthly temperatures vary between 26.7 C and 30.8 C [17]. The studied mosaic consists of 1,800 ha of forest divided into four main blocks (one of them is part of a 13,000-ha forest block that extends beyond the study area), 1,000 ha of actively cultivated rubber groves, abandoned rubber groves overgrown with pioneer species, riparian forest corridors (30–50 m wide and up to 12 km long), cattail wetlands, streams and small rivers. The maximum distance between forest blocks is 1 km and the average distance between riparian corridors is ≤400 m. Although all of the forests were exploited for timber, firewood collecting, and *Euterpe edulis* palm extraction prior to the creation of the reserve in 2005, and some forest patches are manioc fallows, the flora is still highly diverse [18]. The small forest fragments and riparian forests are dominated by pioneer trees (principally, *Cecropia*, *Schefflera*, *Byrsonima*, *Miconia*, *Henrietta*, *Tibouchina*, *Tapirira*, *Senna*, *Kielmeyera*, *Himatanthus*, *Inga*, *Stryphnodendron*, *Bauhinia*), Rubiaceae, Melastomataceae, and Piperaceae shrubs/bushes, thin vines, and *Cyperus* sedges.

**Fig 2:**
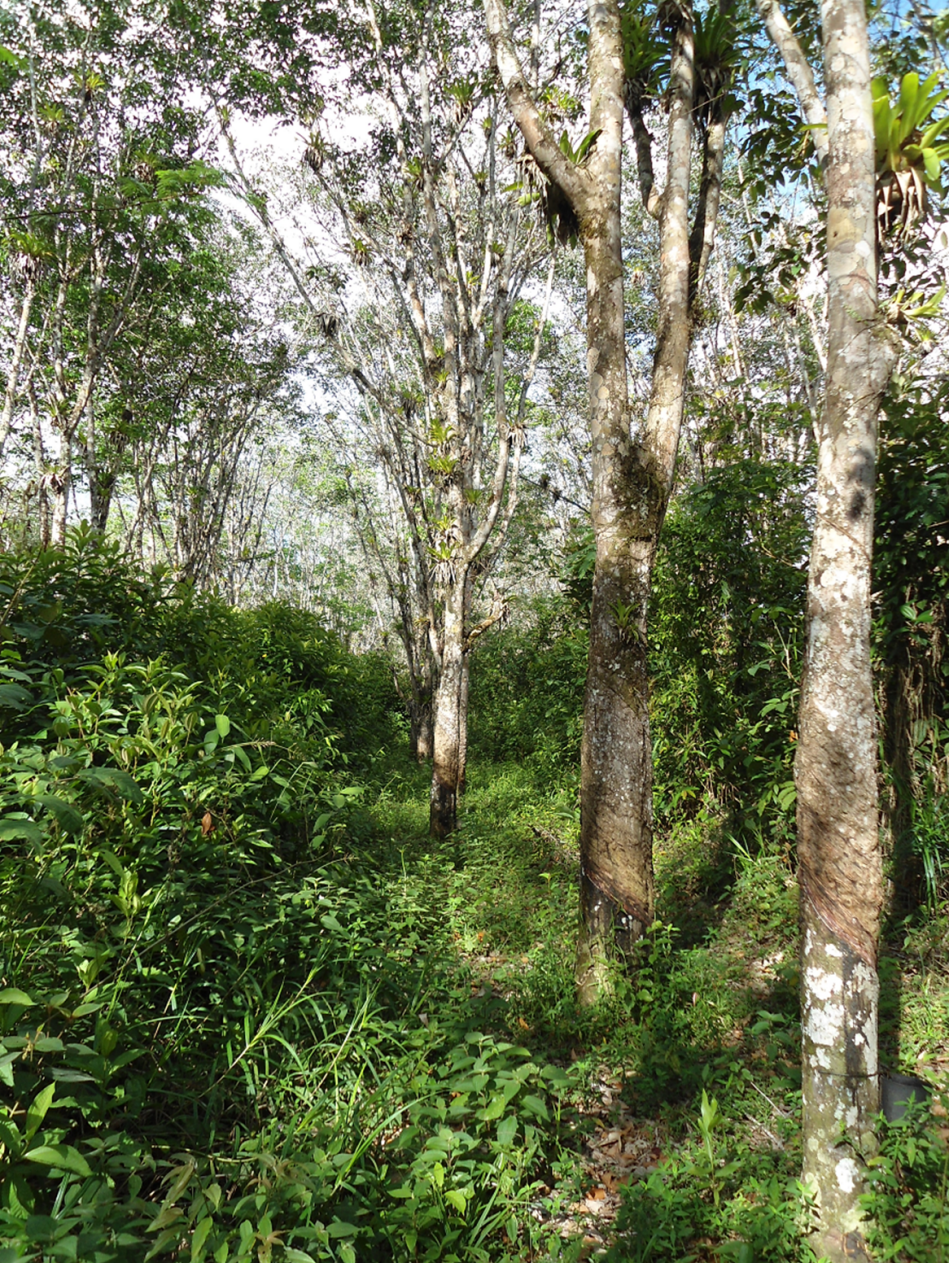
Rubber trees as tall as 15 m with medium-developed inter-rows.

Rubber trees grow up to 15m and are planted in rows with a spacing of 8m (between rows) x 3m (between trees) (Fig 2). This allows a density of approximately 500 trees/ha with pioneer vegetation in the inter-rows. The floristic composition in well-developed inter-rows is similar to that of the riparian forests. Rubber tapping involves a diagonal cut in the bark from which the latex drips into a cup that is attached to the tree. Workers enter the groves by 5:30 a.m. and leave by 12:00 p.m., tapping 900 trees/day with each tree tapped on a 4-day rotation. This schedule is followed year-round. Rubber trees can be tapped for years by shifting the cuttings around the trunk so that, once planted, the landscape is stable for several decades. None of the areas sampled had been replanted since the creation of the plantation in 1950s.

### The study fauna

The study fauna included all of the terrestrial and scansorial mammalian carnivores registered for the region: puma (*Puma concolor*), jaguarundi (*Puma yagouaroundi*), ocelot (*Leopardus pardalis*), margay (*Leopardus wiedii*), oncilla (*Leopardus tigrinus*), tayra (*Eira barbara*), South American coati (*Nasua nasua*), crab-eating raccoon (*Procyon cancrivorus*), crab-eating fox (*Cerdocyon thous*), domestic dog (*Canis familiaris*) and domestic cat (*Felis catus*) [17]. The domestic dog and the domestic cat are the only non-native carnivore species and it is estimated that nearly 70% of the rural households in the study area have dogs and/or cats (A. Dechner, personal communication). All species are mesocarnivores except for the puma. Other species of carnivores found in the landscape but not included in the study because the sampling methods were not adequate to capture their habitat use were: the southern river otter (*Lontra longicaudis*) and the kinkajou (*Potos flavus*). The jaguar was extirpated by hunters during the land conversion events of the 1950/60s [16]. The bush dog (*Speothos venaticus*) may have been historically present in the area [19] but further information on causes and approximate time of disappearance is unclear. In terms of prey species, three terrestrial species of mammals were extirpated between the 19^th^ century and the early 20^th^ century: lowland tapir (*Tapirus terrestris*), white-lipped peccary (*Tayassu pecari*) and giant armadillo (*Priodontes maximus*) (for full list of extant medium and large mammals see [16]). Long-term monitoring (1997–2015) through transect censusing showed that the relative abundance of the medium and large mammal fauna in our study area has increased by 117% since the creation of the REM in 2005 and the implementation of guard patrols (K.M. Flesher, personal communication), with the abundance of prey species reaching levels similar to well-protected reserves elsewhere in the neotropics [16].

### Sampling design and data collection

We set up a 90-site grid with a spacing of approximately 600 m between sampling sites. Of the 90 sampling sites, 30 were in forests, 30 in riparian corridors and 30 in rubber groves (with medium to well-developed inter-rows, i.e. height > 2 m) (Fig 2). We used a trimestral random rotation sampling design in which 30 camera traps were placed at 30 randomly chosen sites (10 in forest, 10 in riparian corridors and 10 in rubber groves) each month, with a subsequent rotation to another 30 sites in the next month and so on until all 90 sites were sampled before repeating any. Thus, each of the 90 sites was sampled four times (once per trimester) between February 2013 and January 2014. Each camera was in place for an average of 22 days/month. The objective of the rotation was to maximize the sampled area, to reduce bait habituation by maximizing the time between bait placement, and to reduce spatial autocorrelation by maximizing the distance between sites sampled at any given time. We conducted this research with approval of the Animal Care and Use committee (IACUC) of Michigan State University under the AUF # 11/12-208-00. In addition, we collected the data with approval of the Brazilian government under the CNPq permit # 001308/2012-2.

We used two brands of cameras, Bushnell (Trophy Cam—Bushnell Corporation—Overland Park, KS) and Tigrinus (4.0D and 6.0D Tigrinus Equipamentos para Pesquisa Ltda—Santa Catarina, SC). Cameras were tested before the study began to gain familiarity with the different programming options offered by each brand and model. Our objectives were to 1) program each camera with equivalent settings (e.g. comparable time intervals between pictures), 2) maximize the chances of capturing all the animals that passed in front of all cameras (i.e. high sensitivity, short interval times between pictures), and 3) minimize possible differences between brands due to improper programming (Table 1). The cameras were randomly assigned to each site while guaranteeing that all sampling sites received both brands over the year. Given that the grid was laid out without reference to previous information on carnivore movements/presence and with the aim that the animals would pass in front of the cameras, we placed lures and baits one time at the beginning of the month at each one of the 30 sites to be sampled. We used a mix of scent lures and dead baits to attract all the different species in the carnivore community [20]. The lures consisted of 500 gr of mashed sardines in oil placed on the ground, 5 ml of banana oil applied on top of a stick placed on one side of the sardines, and 10 ml of a mix of lures (Raccoon Lure No. 1 by Russ Carman, Magna Gland Lure by Russ Carman, Mega Musk Lure by Russ Carman and Ausable Brand Catnip Oil Pint) applied on top of another stick placed on the other side of the sardines. All cameras were placed at a height of 25–35 cm and set to work 24 hours/day.

**Table 1:** Information about the camera traps and programming used.

Bra3nd/Model	Tigrinus 4.0D		Tigrinus 6.0D		Bushnell Trophy cam (XLT & HD)	
	Options available	Selected option	Options available	Selected option	Options available	Selected option
Infrared based-motion sensor	Medium High	High	0–70	8^1^	Low Medium High	Medium^2^
Warm-up sensors^3^	NA	NA	0–70	8	NA	NA
Environment sensor^4^	Medium High	Medium	0–70	35^5^	NA	NA
Time interval between pictures	30 seconds 2 minutes 4 minutes 16 minutes	30 seconds (minimum available)	10 seconds to 99 minutes	10 seconds	1 second to 60 minutes	10 seconds
Flash	Incandescent		Incandescent		Infrared	
Total number of cameras available for rotation	5		27		14	

1. The lower the value the higher the sensitivity. The manufacturer recommends 4–12 for small rodents.

2. Given the observed extremely high sensitivity of the Bushnell cameras in preliminary tests, and in order to reduce false triggers caused by the movements of leaves, we chose to use the normal/medium sensitivity for this brand.

3. Turn on the camera when there is movement in surrounding areas. The lower the value the higher the sensitivity. After activation of these sensors cameras were programmed to be active for 12 seconds.

4. Reduces false triggers caused by environmental factors (e.g. temperature), by blocking the camera for a period. The lower the value the higher the sensitivity. High sensitivity is recommended to reduce false triggers in open areas.

5. After activation of this sensor the camera was blocked for 3 seconds.

### Statistical analysis

To determine the habitat use of a carnivore community, and the effect that different brands of camera traps may have had on their detection, we used a Bayesian approach and a multispecies occupancy model with known species richness [21]. We fit a single-season model due to our limited number of sampling periods (i.e. 4). Occupancy has been defined as the proportion of area, patches, or sample units that is occupied or where a species is present [22]. Occupancy models can be described as a hierarchical, coupled logistic regression, with one regression describing the true occurrence (i.e. state or ecological process) and the other describing the detection probability (i.e. observation process), given that the species occurs in an area [23]. The effects of ignoring imperfect detection (i.e. assuming that a non-detection is equivalent to an absence) have been broadly discussed [24], and occupancy models represent a step forward in ecological studies by estimating occupancy while accounting for imperfect detection.

By including the parameters at the species level as random effects governed by a common set of hyper-parameters or parameters at the community level, multispecies occupancy models allow for the estimation of covariate effects at the species level and of the aggregated effects at the community level (with both levels being related to each other) [25]. These models are valuable given that they increase the precision in occupancy estimates for most species in the community by borrowing strength from the entire community [26]. This allows for the inclusion of infrequently observed species, whose occupancy otherwise could not be estimated through single species occupancy models [25].

Camera data results were organized per species, in a 90 sites (rows) by 4 periods (columns) matrix, containing binary data where 1 represented when a species was detected at a specific site during a specific sampling period and 0 when it was not detected. Thus, we used a binary state model in which true occurrence *z*_*ij*_ is a latent state variable, specified as *z*_*ij*_ *~ Bernoulli (ψ*_*ij*_*)*, where *ψ*_*ij*_ is the occurrence probability of species *i* at site *j*. The observation model was specified as *x*_*ijk*_ *~ Bernoulli (ρ*_*ijk*_*·z*_*ij*_*)*, where *x*_*ijk*_ is the detection/non-detection data for species *i* at site *j* and for the period *k*, and *ρ*_*ijk*_ is the detection probability for species *i* at site *j* and for the period *k* [25]. We included in the occupancy model the different types of habitat as covariates, and in the detection model the different brands of camera traps used. In both models, the covariates were included as indicators (i.e. 0 or 1). To avoid overparameterization, the ecological process model was specified as follows:

$$\mathrm{logit}\;(\psi_{ij})=\alpha_{0i}+\alpha_{1i}forest_{j}+\alpha_{2i}riparian_{j}$$

Where, *ψ*_*ij*_ is the probability that species *i* occurs at site *j*, *α*_*0i*_ is the occurrence probability for species *i* in rubber groves on a logit scale, *α*_*1i*_ is the difference (on a logit scale) in the occupancy probability between rubber groves and forest areas for species *i*, and *α*_*2i*_ is the difference (on a logit scale) in the occupancy probability between rubber groves and riparian areas for species *i*.

The observation model was as follows:

$$\mathrm{logit}\;(\rho_{ijk})=\beta_{0i}+\beta_{1i}camera_{jk}$$

Where: *ρ*_*ijk*_ is the detection probability of species *i* at site *j* for the period *k* (if present, *z* = 1), *β*_*0i*_ is the detection probability (on a logit scale) of species *i* when using Bushnell cameras, and *β*_*1i*_ is the difference in the detection probability (on a logit scale) between Bushnell and Tigrinus cameras for species *i* (S1–S3 Supporting information).

We assumed a closed community (i.e. absence of local extinctions or colonization during one year); however, in order to account for the possibility of weak temporary emigration, and since it may have been confounded with the detection probability, we refer to the occupancy probability as the probability of use sometime during the study period as suggested by Kéry et al. [23]. We did all analyses using R [27] and R2OpenBUGS [28]. We used non-informative priors, and ran three chains each for 55,000 iterations, burning 15,000 and thinning the model by 3. We assessed convergence of the model by looking at the R-hat values, defined as the potential scale reduction factors [28], with values <1.1 being considered acceptable [29]. We tested the prior sensitivity through comparison with model results obtained with different priors. Finally, we assessed model fit by estimating the Bayesian p-value as calculated in Zipkin et al. [25]. The Bayesian p-value is a measure of the discrepancy between the observed data and data simulated under the proposed model, with values close to 0.50 indicating adequate fit and values near 0 or 1 indicating poor fit.

Finally, we explored the spatial autocorrelation between sampling sites, by computing experimental variograms [30]. Given that the locations of the cameras changed every month, we computed one variogram per month with all species’ captures aggregated to increase the number of sites with observations. Although experimental variograms do not require that the data follow a specific distribution [30], it has been demonstrated that asymmetric data, particularly in analyses with small sample sizes, can result in unreliable variograms [31]. Considering that our data are asymmetric, and only for the purpose of computing the variograms, we square-root transformed our count data to reduce the positive skewness cause by a large number of zeroes [32]. Variograms were calculated using the R package GeoR [33]. To observe how the semivariance changed as a function of the predetermined distances in the grid, we created lag intervals of 600 m (equivalent to the grid cell size). We set up a maximum lag distance of 4,800 m (roughly one third of the largest distance between pairs [30]).

## Results

We registered 483 captures (a species detection/24 hrs.) in 7,954 camera days (Bushnell = 3,203, Tigrinus = 4,751) (Forest = 2,849, Riparian Corridors = 2,370, Rubber Groves = 2,735) (Table 2). The species with the highest numbers of captures were coati (n = 208), domestic dog (n = 144) and crab-eating fox (n = 79). For the statistical analysis, we merged the captures of ocelot, margay and the unidentified individuals of the genus *Leopardus* into one group (referred hereafter as wildcats *Leopardus* spp.). We did so because the low number of captures per each species (≤10) would generate extremely sparse data. We excluded the registrations of puma and raccoon from the analyses due to the low number of captures for these species. The only species inhabiting the landscape that were not registered by the cameras were the domestic cat and the jaguarundi, although their presence was confirmed through direct sightings.

**Table 2:** Total number of captures and sites where each species was detected per habitat in 7,954 camera days, Ituberá/Igrapiúna, Bahia, Brazil, 2013–2014.

Species	Common name	Forest		Riparian		Rubber		Total	
		Captures	Sites	Captures	Sites	Captures	Sites	Captures	Sites
*Canis familiaris*	Domestic dog	16	7	19	13	109	24	144	44
*Cerdocyon thous*	Crab-eating fox	2	2	19	14	58	21	79	37
*Eira barbara*	Tayra	8	6	12	10	6	5	26	21
*Leopardus pardalis*	Ocelot	1	1	0	0	4	2	5	3
*Leopardus wiedii*	Margay	2	2	4	4	4	2	10	8
*Leopardus spp*.	(unidentified)	5	4	2	2	0	0	7	6
*Nasua nasua*	South-American coati	156	28	48	20	4	3	208	51
*Procyon cancrivorus*	Crab-eating raccoon	0	0	2	1	0	0	2	1
*Puma concolor*	Puma	1	1	1	1	0	0	2	2
Total		191	51	107	65	185	57	483	173

**Fig 3:**
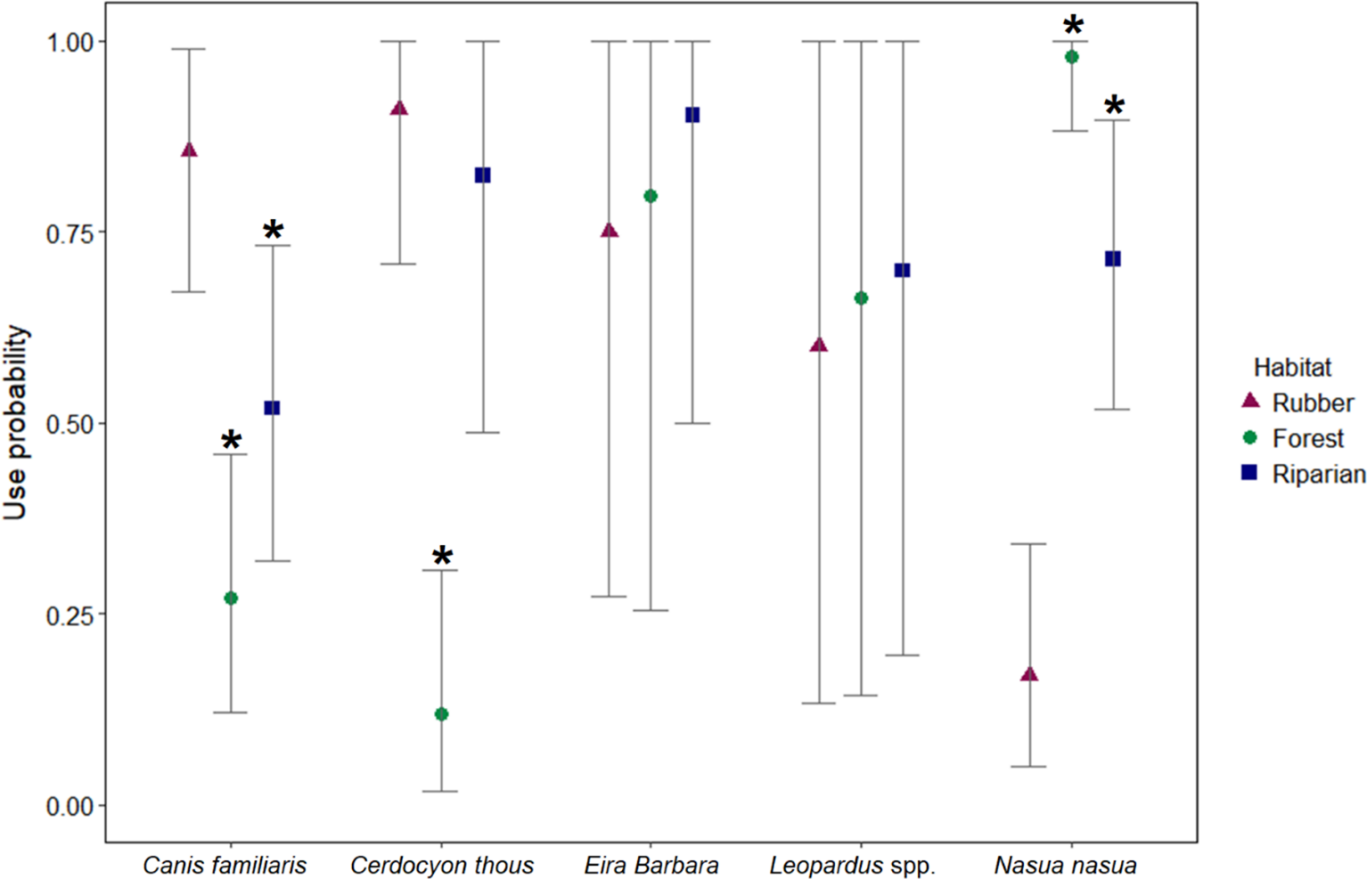
Probabilities of habitat use by each species, Ituberá/Igrapiúna, Bahia, Brazil, 2013–2014. The symbols indicate the posterior means and the bars indicate the 95% posterior intervals. The star indicates significant differences between the use of each habitat in comparison with the use of rubber areas (baseline in model).

Results from the multispecies occupancy model showed that although the average number of sites used at least once during the study period for most of species was similar (Table 3), there were large differences in terms of the habitats they used (Fig 3). Coatis showed a significantly higher probability of using forest than rubber groves (*α*_1Coati_ = 8.76, 95% posterior interval = 3.41 to 25.64), and a significantly higher probability of using riparian corridors than rubber groves (*α*_2Coati_ = 2.69, 95% posterior interval = 1.24 to 4.38). Domestic dogs showed a higher probability of using rubber groves than forest sites, and to a lesser extent, than riparian corridors (Fig 3). The differences between the use of rubber groves and forest, and between rubber groves and riparian corridors were in both cases statistically significant, given that the 95% posterior interval of these parameters did not overlap zero (*α*_1dog_ = -3.06, 95% posterior interval = -5.61 to -1.45, and, *α*_2dog_ = -1.94, 95% posterior interval = -4.50 to -0.33, respectively).

**Table 3:** Posterior estimations of carnivore occupancy and detection, Ituberá/Igrapiúna, Bahia, Brazil, 2013–2014. PI = Posterior intervals.

		# of sites occupied	Mean ψ	Mean ψ forest	Mean ψ riparian	Mean ψ rubber	Mean ρ	Mean ρ Bushnell	Mean ρ Tigrinus
Domestic dog (*Canis familiaris*)									
	Mean	49.59	0.55	0.27	0.52	0.85	0.41	0.54	0.35
	SD	2.97	0.06	0.09	0.11	0.08	0.04–0.00	0.07	0.05
	95% PI	45–56	0.44–0.66	0.12–0.46	0.32–0.73	0.67–0.99	0.33–0.5	0.40–0.68	0.27–0.45
Crab-eating fox (*Cerdocyon thous*)									
	Mean	55.96	0.62	0.12	0.82	0.91	0.23	0.37	0.15
	SD	6.13	0.08	0.08	0.16	0.08	0.04	0.07	0.03
	95% PI	44–66	0.46–0.74	0.02–0.31	0.49–1.00	0.71–1.00	0.16–0.31	0.25–0.52	0.09–0.22
Tayra (*Eira barbara*)									
	Mean	73.63	0.82	0.80	0.90	0.75	0.09	0.18	0.04
	SD	15.03	0.17	0.24	0.14	0.23	0.03	0.06	0.02
	95% PI	38–90	0.42–1.00	0.25–1.00	0.50–1.00	0.27–1.00	0.05–0.17	0.10–0.35	0.02–0.08
Wildcats (*Leopardus* spp.)									
	Mean	58.83	0.65	0.66	0.70	0.60	0.10	0.21	0.05
	SD	23.42	0.26	0.31	0.28	0.29	0.06	0.11	0.03
	95% PI	20–90	0.21–1.00	0.14–1.00	0.20–1.00	0.13–1.00	0.04–0.25	0.08–0.49	0.01–0.14
South American coati (*Nasua nasua*)									
	Mean	55.21	0.62	0.98	0.71	0.17	0.42	0.67	0.29
	SD	2.00	0.04	0.03	0.10	0.08	0.03	0.06	0.04
	95% PI	52–60	0.54–0.70	0.88–1.00	0.52–0.90	0.05–0.34	0.35–0.49	0.56–0.78	0.22–0.37

Crab-eating foxes showed a significantly higher probability of using rubber groves than forest sites (*α*_1fox_ = -5.26, 95% posterior interval = -10.17 to -2.58), and a non-significant difference between the use of rubber groves and riparian corridors (*α*_2fox_ = -0.06, 95% posterior interval = -4.05 to 6.94). For coatis, domestic dogs and foxes, riparian corridors seemed to be of intermediate use between forest and rubber (Fig 3). Tayras and wildcats showed no significant differences in the probabilities of habitat use, and the wide posterior intervals indicated a lack of precision in the estimation of habitat use (Fig 3).

**Fig 4:**
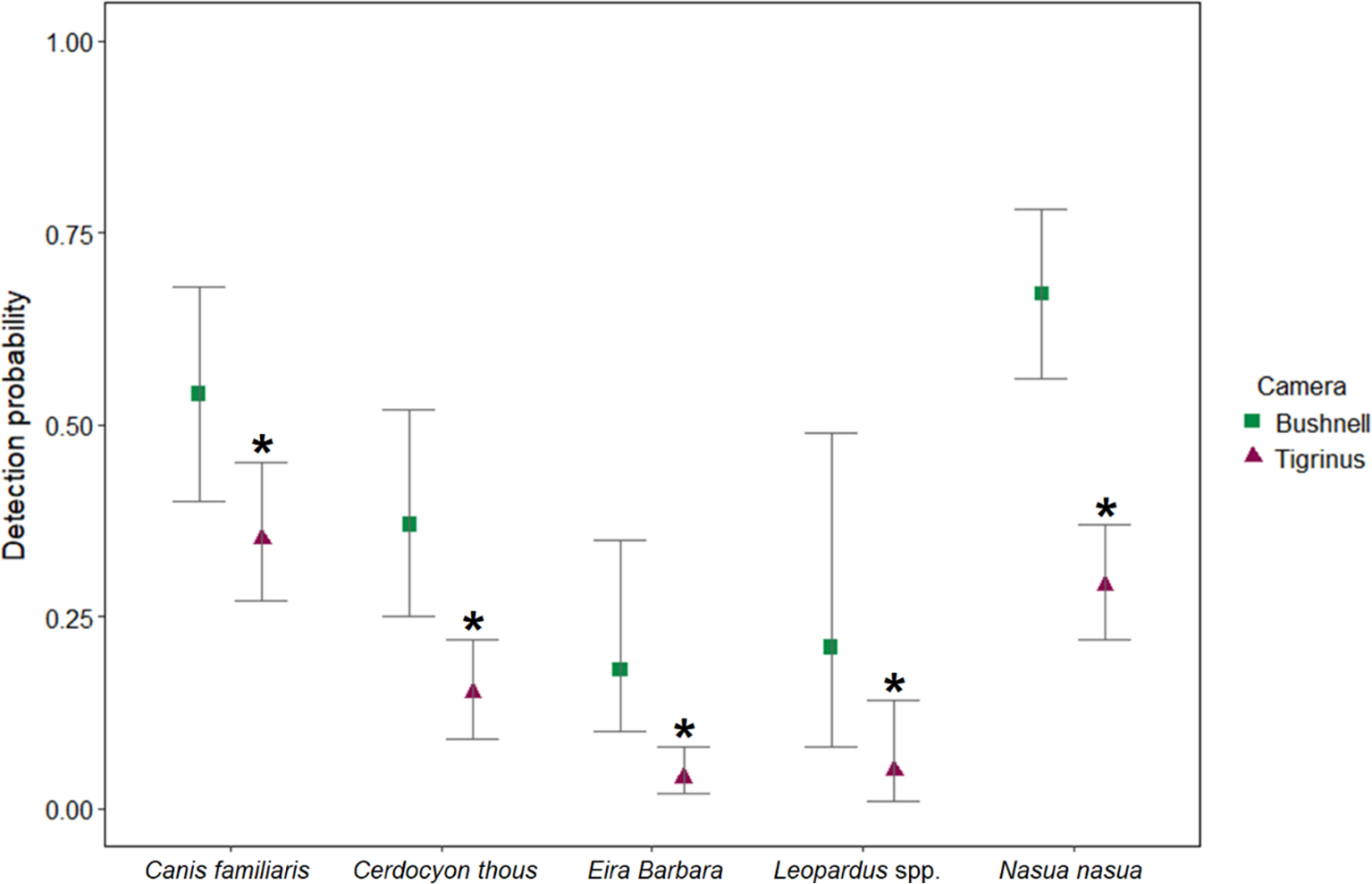
Detection probabilities for each species, Ituberá/Igrapiúna, Bahia, Brazil, 2013–2014. The symbols indicate the posterior means and the bars indicate the 95% posterior intervals. The star indicates significant differences between the detection probability while using Tigrinus in comparison with Bushnell (baseline in model).

The detection probabilities of the studied species ranged between 0.18 and 0.67 when using Bushnell, and between 0.04 and 0.35 when using Tigrinus. Tayras and wildcats had the lowest detection probabilities regardless of the brand used (Table 3). There was a lower detection probability for all species when using Tigrinus as opposed to Bushnell cameras (Fig 4). The difference between the camera brands had a significant effect on the detection probability of all species (*β*_1*Dog*_ = -0.78, 95% posterior interval = -1.40 to -0.12, *β*_1*Fox*_ = -1.23, 95% posterior interval = -1.80 to -0.65, *β*_1*Tayra*_ = -1.69, 95% posterior interval = -2.58 to -0.99, *β*_1*Leop*._ = -1.68, 95% posterior interval = -2.65 to -0.93, *β*_1*Coati*_ = -1.61, 95% posterior interval = -2.21 to -1.08).

The goodness of fit test for the model was 0.53, indicating that the habitat model provided an adequate description of the data. In addition, experimental variograms indicated that the number of pairs of sites in the first lag interval is low (≤15), and that there are not observable spatial coherent structures (S4 Supporting information). Thus, the monthly grid-based rotation of the cameras in our mosaic was effective at maximizing the space between cameras and at controlling spatial autocorrelation.

## Discussion

### Species habitat use in the rubber/forest mosaic

The studied landscape supports a nearly intact mammalian carnivore community seven decades after its conversion from continuous forest to a rubber/forest mosaic. Nonetheless, not all species use the new mosaic in a similar fashion. This differential habitat use by species is important as it not only determines their distribution in the landscape but may modulate their inter and intra-specific interactions [34].

Coatis showed a significantly higher probability of using forests and riparian corridors than rubber groves which is consistent with other studies that have described coatis as a forest species [35–38]. On the other hand, camera trap studies conducted exclusively in neotropical lowland moist forests have frequently recorded few coatis’ captures [39] and/or have reported moderate occupancy values for this species (≤0.42 [40]) compared to our mean occupancy value (0.98 in forests). We believe that the difference may be due to the fact that coatis in those areas may be spending more time in the canopy than on the ground to avoid predation, as the carnivores with the highest capture frequencies and/or occupancy values in those studies were ocelots, jaguars and pumas, all predators of coatis [41]. The relatively high detection probability of coatis, in comparison to other species, was likely because it was the only species that showed a consistently strong attraction towards the lures. Not surprisingly, we found that 91% of the captures of coatis were registered during the day (i.e. from 6:00 a.m. to 5:59 p.m.), and that the difference between day and night captures was statistically significant (one-way chi-square test, *x*^2^ = 138.94, *df* = 1, *p*< 0.001).

Domestic dogs are non-native carnivores but are nonetheless an important part of the ecosystem due to their abundance, widespread distribution, and the fact that they prey on native species throughout the Atlantic forest [42–44]. We found that domestic dogs have significantly lower probabilities of using forests (mean probability of 0.27) than riparian corridors and rubber crops, indicating that the forest may serve as a partial refuge for wildlife from this non-native predator. The preference of dogs for the rubber groves is not surprising as they often accompany their owners during their work in the rubber groves. Although they also enter the forest to pursue prey on their own or in the company of hunters, the higher use probabilities of dogs in the rubber groves and the frequent camera trap registers of dogs with rubber tappers, indicate that dogs generally preferred to stay close to their owners rather than to enter the forest on their own.

There was high spatial overlap in habitat use between dogs and foxes, but their use of these areas was temporally separated; 92% of dog captures were diurnal while 73% of fox captures were nocturnal (two-way chi-square, *x*^2^ = 98.69, *df* = 1, *p*< 0.001). Foxes’ preference for rubber groves and riparian corridors and their low probability of using forest is consistent with the results from other studies showing that foxes prefer open, forest edge and agricultural habitats in mosaic landscapes [2, 35, 36, 45].

The wide posterior intervals for the habitat use probabilities of tayras and wildcats, allowed us to highlight a pattern in the precision of the occupancy parameter of species with low detection probabilities and moderate capture values, which we discuss further in the next section. We found that 96% of the captures of tayras were registered during the day (one-way chi-square test, *x*^2^ = 22.16, *df* = 1, *p*< 0.001), while 96% of the captures of wildcats were registered at night (one-way chi-square test, *x*^2^ = 18.18, *df* = 1, *p*< 0.001). In spite of the fact that we only captured pumas twice, once in the forest and once in a riparian corridor, we regularly found scats, tracks, and rakings in all forest habitats and, less frequently, in the rubber groves. The small size of the study area relative to the home range of pumas suggests that there are few individuals inhabiting the area. The low number of captures may be related to the fact that pumas regularly use trails and unpaved roads [46], and thus the placement of the cameras independent of the trail network may have made our sampling design inadequate for determining the habitat use of this species. The low number of captures of crab-eating raccoons was unexpected as we found tracks of this species and saw it along the roads throughout the landscape. Other scientists in Bahia found raccoons to be common in other agroforestry landscapes [36, 47] and on a larger regional scale raccoons were widely reported and recorded in all landscape types; they are wetland habitat specialists, but generalists in terms of broader habitat classifications (i.e. they exploit wetland habitats wherever these occur in the regional landscape) [17].

There were two terrestrial species that were not captured by the camera traps, the jaguarundi and the domestic cat. We did see the jaguarundi several times in the rubber groves and riparian corridors but sightings were infrequent and interviews in the broader region indicate that the jaguarundi is rarely seen [17]. These results are consistent with other studies in areas with differing levels of human disturbance in which the species was not recorded or recorded with very low capture rates [36, 39, 48]. Domestic cats, although found in human settlements, were not captured in any of our sampled sites which is consistent with other studies in the region where domestic cats were rarely recorded [36], suggesting that cats do not wander far from human settlements.

### Sampling and statistical methods

Camera traps are being increasingly used in ecological studies as they make it possible to collect information on elusive species in a minimally invasive manner and are cost-effective [49, 50]. While today there is a wide selection in the market of camera traps, some studies have shown variability in performance among brands, models [51], and even between cameras of the same model [12]. Protocols for monitoring of birds and mammals recommend the use of one or two high-end models of camera traps, possibly to reduce the bias derived from this variability [52, 53]. However, high-end camera traps are costly. This is critical considering that most biodiversity hotspots are in developing countries where the resources to obtain high-end camera traps may not be readily available.

We found that the detection varied among species and that tayras and wildcats had the lowest detection probabilities. Low detection probabilities make it difficult for occupancy models to distinguish between a site where the species is truly absent and a site where it is simply not detected. Thus, occupancy estimates produced with low detection probabilities (<0.15) are unreliable [24, 54]. We found that there is a significantly higher detection probability for the studied carnivore species when using Bushnell as opposed to Tigrinus cameras. The difference in detection probabilities may be due to differences in sensitivity, trigger speed, and noise produced by the equipment (as it may scare carnivores away). The Tigrinus cameras are composed of a Sony Cyber-shot embedded in a rigid box which, when activated, produces a louder noise than the Bushnell cameras. This difference in the detection probability between the different brands of camera traps show the necessity of accounting and/or controlling for such factors when necessary. This can be done by estimating ecological parameters while accounting for imperfect detection. Although obtaining the data needed to adjust statistically for imperfect detection may require more effort to be able to provide reliable parameter estimations (e.g. extending the sample period to increase detection rates [54, 55]), ignoring the effect of the cameras on the detection probability may seriously bias the results and therefore mislead conservation actions. In terms of sampling, the rotation of the camera traps used in this study proved to be effective at controlling spatial autocorrelation.

**Fig 5:**
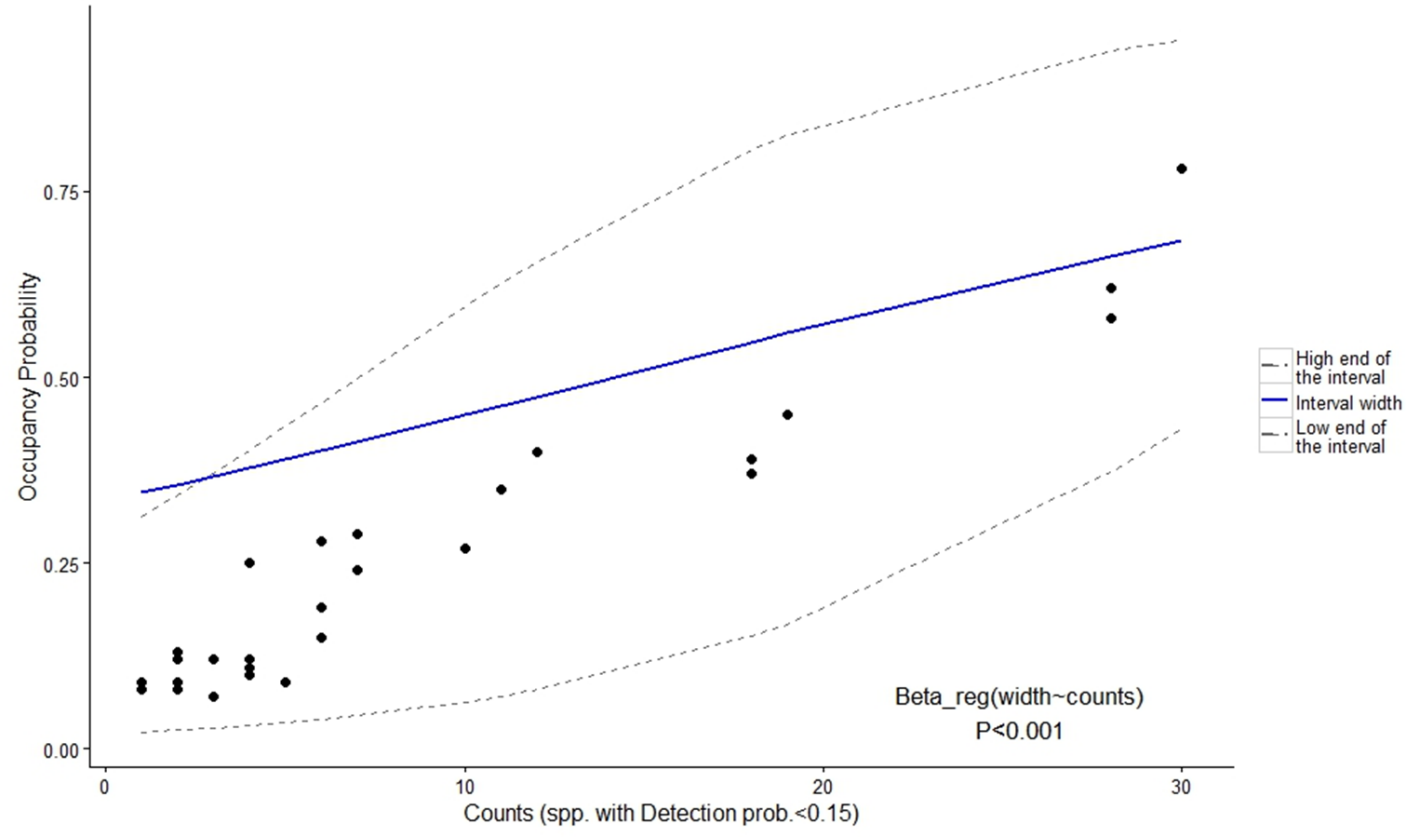
Relationship between the number of detections (counts or captures) and the predicted width, high end, and low end of the 95% interval of the occupancy parameter for species with detection probability <0.15 in a multispecies occupancy model. Data taken from Zipkin et al. 2009.

One of the benefits of multispecies occupancy modeling is derived from the potential these models offer for increasing the precision of coefficients of rarely detected species by borrowing strength from the entire community [26]. However, the wide posterior intervals for habitat use probability, reaching 1.00 in all cases for tayras and wildcats seem to be an artifact of the model. This artifact appears to occur when the species exhibit a moderate level of captures and a low detection probability (Fig 5). This indicates that when using these models, the occupancy estimation of a species that is moderately common but difficult to detect is highly uncertain with a tendency for the model to suggest that the species could be everywhere.

Finally, we call for caution when drawing conclusions about the ecological requirements of carnivore species derived from observations/captures as these always depend on the species detection probabilities. We agree with Banks-Leite et al. [56] that in tropical areas where some species are naturally rare and logistics frequently complex, it can be difficult to implement models that adjust for imperfect detection. However, it is then the ethical obligation of researchers to control the effect of known factors on the detection probability, or to be conservative when drawing conclusions as in many cases there may be unknown factors affecting the species detections. Detection probability may be affected by logistic and environmental factors, as well as by interspecific interactions and by the ecology of the species per se. Providing conclusions about species with low samples sizes, low numbers of sampled sites, low sampling effort, low detection probabilities, or ignoring the fact that sometimes species present higher detection probabilities in disturbed ecosystems (either because of concentrated foraging activities or due to visibility), may lead to erroneous conclusions and as a result lead to inappropriate conservation actions.

Although in this paper we described the habitat use of carnivores after 70-years of conversion from continuous forest to a rubber/forest mosaic, information related to the temporal dynamics of the species, as well as their intra/inter specific interactions is lacking. This information is essential to fully understand the effects of landscape modification on the ecology of the species. In addition, although it is frequently assumed that the capture of an animal in a specific habitat is an indicator of long-term habitat suitability, other factors affecting populations at different temporal and spatial scales, which are usually not considered in ecological studies (e.g. habitat change and its effects on the physical and reproductive health of species), should be considered.

## Management recommendations

The results from this study along with the characteristics of our study area, provide information that is applicable to the conservation of carnivores in rubber/forest landscapes:

- The forest and the riparian corridors set aside when the plantation was created (31% of the plantation), have proved to be sufficient for the carnivores to persist for seven decades (with exception of the jaguar). The study area is clearly not large enough to sustain viable populations of the puma and perhaps several of the other species (e.g. ocelots, tayras), but given that the regional landscape retains 40% forest cover, the rubber/forest mosaic continues to play an important role in sustaining these species at the regional scale.
- Maintaining riparian forests is important as our results showed that these are supplementary habitat for most species.
- Given that the floristic composition in well-developed inter-rows of rubber plantations is similar to that in riparian areas [17], and in turn, that riparian areas have an intermediate use probability for most carnivore species, managers should allow pioneer vegetation in the rubber inter-rows to grow as this increases plant diversity in the rubber monocultures. In addition, some species may find adequate food resources in the pioneer vegetation that characterizes these areas (e.g. consumption of fruits of *Henrietta succosa* sp. by foxes; A. Dechner, personal observation); thus allowing the vegetation in the inter-rows to grow could increase the food resources available to carnivores in rubber grows.
- Domestic dogs have a negative impact on other carnivores and on wildlife in general [42, 44], thus they can influence the capacity of other species to use the rubber plantations and to move throughout the greater landscape. Considering this and given the integration of dogs in the local communities, management plans to reduce the negative impact that domestic dogs have on wildlife should be explored and implemented in rubber plantations.
- Given that in our study area the relative abundance of the medium and large mammal fauna has increased by 117% after the implementation of guard patrols in 2005 (K.M. Flesher, personal communication), and that our results were therefore conditioned to this patrolling system, hunting should be prohibited and the ban enforced in the plantations, as this will increase the probability of maintaining carnivores in these areas.

This study shows that most carnivores can persist on rubber plantations if sufficient forest cover is retained. By adopting sustainable practices (e.g. creating forest reserves and riparian corridors) it is possible to make rubber plantations carnivore friendly. Preserving the largest cats (e.g. jaguars, tigers) is more complicated due to potential conflict with humans and it is likely that only by retaining large areas of natural forest will these species survive.

## Supporting information

S1 Supporting information R code for fitted model. (R)

S2 Supporting information Species data. (CSV)

S3 Supporting information Covariates data. (CSV)

S4 Supporting information Experimental spatial variograms. (DOCX)
