# Correction: Determining carnivore habitat use in a rubber/forest landscape in Brazil using multispecies occupancy models

**DOI:** 10.1371/journal.pone.0197397

**Published:** 2018-05-09

**Authors:** Andrea Dechner, Kevin M. Flesher, Catherine Lindell, Téo Veiga de Oliveira, Brian A. Maurer

The fourth author's name is spelled incorrectly. The correct name is: Téo Veiga de Oliveira. The correct citation is: Dechner A, Flesher KM, Lindell C, Veiga de Oliveira T, Maurer BA (2018) Determining carnivore habitat use in a rubber/forest landscape in Brazil using multispecies occupancy models. PLoS ONE 13(4): e0195311. https://doi.org/10.1371/journal.pone.0195311
