# Supplementary material for: Determining carnivore habitat use in a rubber/forest landscape in Brazil using multispecies occupancy models
Source: PLoS One. 2018 Apr 16;13(4):e0195311. doi: 10.1371/journal.pone.0195311 (PMC5901926; doi:10.1371/journal.pone.0195311)

**S4 Supporting information**. Experimental spatial variograms of the sampling sites selected from a monthly grid-based rotation. Lag interval is 600 m (equivalent to the grid cell size). Maximum lag distance is 4,800 m (roughly one third of the largest distance between pairs).


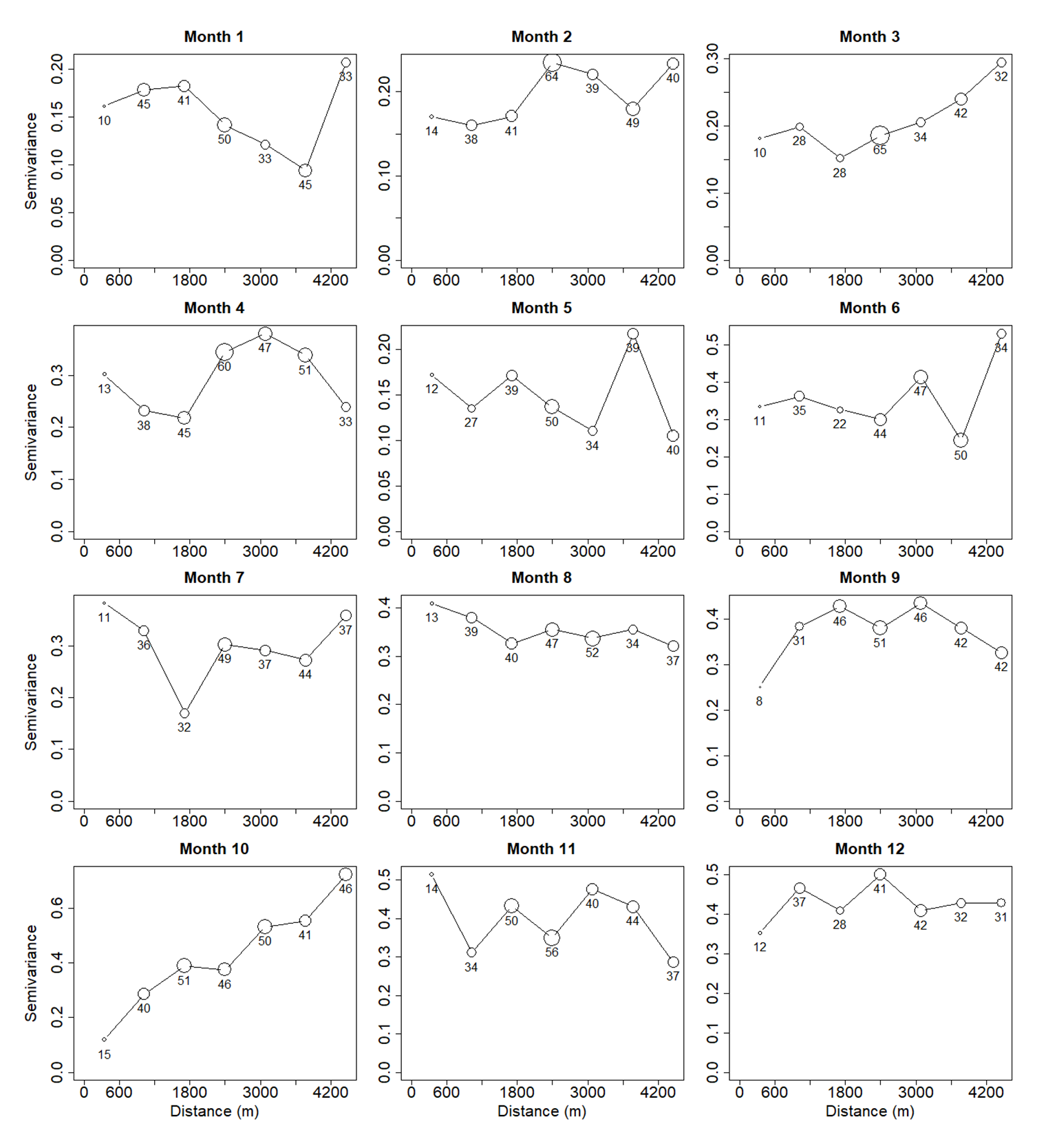

Supplement: S4 Supporting information — (DOCX) [file pone.0195311.s004.docx]
